# Postmarketing safety of migraine prophylactic monoclonal antibodies: An EudraVigilance database analysis of eptinezumab, fremanezumab, galcanezumab, and erenumab

**DOI:** 10.1111/head.14962

**Published:** 2025-05-29

**Authors:** Victoria Nikitina, Greta Santi Laurini, Nicola Montanaro, Domenico Motola

**Affiliations:** ^1^ Unit of Pharmacology, Department of Medical and Surgical Sciences Alma Mater Studiorum University of Bologna Bologna Italy; ^2^ Alma Mater Studiorum University of Bologna Bologna Italy

**Keywords:** anti‐calcitonin gene‐related peptide monoclonal antibodies, migraine, pharmacovigilance, prophylaxis of migraine, safety

## Abstract

**Objectives/Background:**

This study was undertaken to evaluate the postmarketing safety of monoclonal antibodies (mAbs) targeting the calcitonin gene‐related peptide pathway used for migraine prophylaxis through pharmacovigilance data analysis by examining suspected adverse events reported in Europe. Migraine is one of the most prevalent and debilitating neurological disorders globally. The introduction of mAbs targeting the calcitonin gene‐related peptide pathway has transformed migraine prophylaxis. However, their safety profiles in real‐world settings are not fully established, and ongoing safety monitoring is essential, as clinical trials may not capture all potential adverse drug reactions associated with new therapies.

**Methods:**

A disproportionality analysis was carried out by analyzing postmarketing pharmacovigilance data from the EudraVigilance database, focusing on four mAbs: eptinezumab, fremanezumab, galcanezumab, and erenumab. Descriptive and statistical analyses were performed on data retrieved from the date of marketing authorization of each medicine up to June 16, 2024. The reporting odds ratio (ROR), information component, and empirical Bayes geometric mean were calculated to detect signals of disproportionate reporting comparing their safety profiles to topiramate, a standard preventive migraine treatment.

**Results:**

A total of 14,285 reports had emerged; most of them were from females and concerned patients aged 18–64 years. The most frequently reported adverse drug reactions were primarily nonserious, aligning with literature and previously established safety profiles, such as fatigue and injection site reactions. The statistical analysis revealed 15 significant disproportionality signals: 11 for eptinezumab and four for galcanezumab. Eptinezumab highlighted potential new safety signals such as palpitations (ROR = 6.93, 95% confidence interval [CI] = 3.39–14.18), oropharyngeal pain (ROR = 7.19, 95% CI = 3.40–15.24), and erythema (ROR = 12.31, 95% CI = 4.58–33.12). These findings suggest a potential class effect, warranting further investigations and highlighting the importance of continued monitoring regarding the long‐term safety of mAbs.

**Conclusion:**

Almost all of the most reported and statistically significant adverse events were nonserious and consistent with the existing literature. Given the chronic nature of migraine treatment, continuous pharmacovigilance monitoring is essential to ensure their constant safe use in clinical practice.

## Plain Language Summary

Although monoclonal antibodies targeting the calcitonin gene‐related peptide pathway have greatly improved migraine prevention, their long‐term safety in real‐world settings is still not fully understood. In this study, we analyzed postmarketing safety data from the European database EudraVigilance, with a focus on significant adverse events related to monoclonal antibody use. The findings showed that most reported side effects were not serious and were in line with previous studies, but ongoing safety monitoring for these treatments in clinical practice is important.

**Abbreviations**
ADR: adverse drug reaction
AE: adverse event
CGRP: calcitonin gene‐related peptide
CI: confidence interval
EBGM: empirical Bayes geometric mean
EMA: European Medicines Agency
EU: European Union
EV: EudraVigilance
FAERS: FDA Adverse Event Reporting System
IC: information component
ICSR: Individual Case Safety Report
IME: Important Medical Event
mAb: monoclonal antibody
MedDRA: Medical Dictionary for Regulatory Activities
PT: Preferred Term
ROR: reporting odds ratio
SPC: Summary of the Product Characteristics

## INTRODUCTION

Pharmacovigilance contributes to public health protection, playing a crucial role in ensuring the postmarketing safety of medicines. The World Health Organization defines pharmacovigilance as a science and activities relating to the detection, assessment, understanding, and prevention of adverse effects or any other medicine/vaccine‐related problem.^1^ Spontaneous reporting systems, such as that of EudraVigilance (EV), play a key role in pharmacovigilance by allowing the early detection of potential safety signals and ongoing assessment of potential risks related to reported adverse drug reactions (ADRs).^2^ It is especially important for identifying rare or long‐term ADRs that might not be observed in premarketing clinical trials, which are limited in duration and number of participants, and conducted in strictly controlled settings.^3^, ^4^, ^5^ The data are collected from various sources, such as different categories of health care professionals and also patients, and this allows providing insights into unknown ADRs and emerging safety concerns in real clinical practice. Postmarketing data reflect real‐world patient populations, as they include those excluded from clinical trials, such as vulnerable groups and patients with comorbidities and/or with multiple medications.

This is important for all drugs on the market, particularly those that have recently come onto the market, such as monoclonal antibodies (mAbs) used in migraine prophylaxis. Migraine is one of the most prevalent and debilitating neurological disorders globally, and its pharmacological treatments are typically categorized into acute and preventive strategies.

mAbs targeting the calcitonin gene‐related peptide (CGRP) pathway, such as eptinezumab, fremanezumab, galcanezumab, and erenumab, have emerged as a significant advancement in migraine prophylaxis (Table 1 illustrates their mechanisms of action). CGRP is a neuropeptide that modulates nociceptive signaling and acts as a vasodilator, playing a crucial role in migraine pathophysiology. Unlike other neuropeptides, CGRP levels increase significantly during a migraine attack and return to normal upon headache relief.

**TABLE 1:** Monoclonal antibodies for the prophylaxis of migraine approved in the European Union.

Active substance	Therapeutic indications	Mechanism of action	Date of first authorization
Eptinezumab	Eptinezumab is indicated for the prophylaxis of migraine in adults who have at least 4 migraine days per month.	Eptinezumab is a recombinant humanized IgG1 antibody that binds to α and β forms of human CGRP ligand. It prevents the activation of CGRP receptors and hence the downstream cascade of physiological events linked to initiation of migraine attacks. Eptinezumab inhibits α‐ and β‐CGRP‐mediated neurogenic inflammation and vasodilation.	January 24, 2022
Erenumab	Erenumab is indicated for prophylaxis of migraine in adults who have at least 4 migraine days per month.	Erenumab is a human monoclonal antibody that binds to the receptor. It potently and specifically competes with the binding of CGRP and inhibits its function at the CGRP receptor.	July 26, 2018
Fremanezumab	Fremanezumab is indicated for prophylaxis of migraine in adults who have at least 4 migraine days per month.	Fremanezumab is a humanized IgG2Δa/kappa monoclonal antibody. It selectively binds the CGRP ligand and blocks both CGRP isoforms (α‐ and β‐CGRP) from binding to the CGRP receptor.	March 28, 2019
Galcanezumab	Galcanezumab is indicated for the prophylaxis of migraine in adults who have at least 4 migraine days per month.	Galcanezumab is a humanized IgG4 monoclonal antibody that binds CGRP, thus preventing its biological activity.	November 14, 2018

Abbreviations: CGRP, calcitonin gene‐related peptide; IgG1, immunoglobulin G1.

Clinical trials and real‐world studies have provided valuable insights into the safety profiles of these mAbs; the known and common ADRs include hypersensitivity reactions such as rash, pruritus, and urticaria and general disorders, and administration site conditions such as injection site reactions. Despite their proven efficacy in clinical trials, the long‐term safety of medicines necessitates rigorous postmarketing surveillance to monitor safety and identify potential safety signals that may not have been evident during clinical trials, especially given the chronic nature of migraine treatment.

Therefore, the aim of this study was to investigate an overall postmarketing safety profile of eptinezumab, fremanezumab, galcanezumab, and erenumab in Europe.

## METHODS

### Study design and data source

This is a retrospective pharmacovigilance study, based on disproportionality analysis, conducted on data gathered from the European Union (EU) postmarketing surveillance database EV via the adrreports.eu online interface.^6^

EV is a publicly accessible spontaneous reporting system managed by the European Medicines Agency (EMA) for the EU. It collects Individual Case Safety Reports (ICSRs) concerning suspected adverse drug reactions across the European Economic Area.^7^ In large databases like EV, it is crucial to remove duplicates. Therefore, the EMA has a specific process of detecting and managing duplicates during periodic screenings. An initial grouping of ICSRs is performed based on the primary source country and sex and age of the patient. The EV algorithm further quantifies the difference of ICSRs from a statistical point of view taking into account additional parameters related to the patient, the primary source, and the reported medicinal product(s)/active substance(s) and adverse reaction(s), as well as the fact that case information may vary, for example, due to differences in coding practices.^8^ The research included all reports related to the four mAbs reported as suspected drug, including cases where the brand name was not specified: eptinezumab, erenumab, fremanezumab, and galcanezumab.

### Study period

The study period extended from the date of marketing authorization of each medicine (Table 1) up to June 16, 2024.

### Statistical methods

Both descriptive and statistical disproportionality analyses were conducted on reported suspected adverse events (AEs).

The primary analysis focused on identifying potential safety signals through the disproportionality analysis method reporting odds ratio (ROR) with 95% confidence interval (CI). Two other statistical analysis methods, information component (IC) and empirical Bayes geometric mean (EBGM), were performed to ensure the robustness of the results. For the manipulation of data, calculation of disproportionality measures, and visualization of results, Excel was used (software developed by Microsoft).

### Descriptive analysis

We performed a descriptive analysis of all the reports related to the mAbs included in this study. The extracted ICSRs were all spontaneous, identified by a unique EU Local Number, reporting information on primary source qualification (health care professional or non‐health care professional), EV gateway receipt date, patient sex and age group, Medical Dictionary for Regulatory Activities (MedDRA) Preferred Terms (PTs), seriousness criteria, and suspect and concomitant drugs. The EV database provides information on the sex of patients by dividing them into three categories: female, male, or unknown. Gender information is not present in EV. The terminology used for coding the AEs was MedDRA, a standardized medical terminology developed to facilitate sharing of regulatory information internationally for medical products. The MedDRA terminology is organized in a five‐level multiaxial hierarchy, offering greater specificity as you go down the levels.^9^ At the top, there are 27 System Organ Classes, which encompass High Level Group Terms and High Level Terms at the subsequent levels. Each next level member (PT) represents a unique descriptor (a single medical concept) for symptoms, signs, disease diagnoses, therapeutic indications, investigations, surgical or medical procedures, and medical social or family history traits. Finally, each PT is associated with one or more Lower Level Terms; these Lower Level Terms are essentially entry terms that include synonyms and lexical variants.^10^, ^11^, ^12^ Each EV report may include one or more symptoms. AEs were classified as serious based on the Important Medical Event (IME) list version 27.0. For each drug, the notoriety of reported AEs was assessed according to the respective Summary of Product Characteristics (SPC).^13^, ^14^, ^15^, ^16^

### Disproportionality analysis

The statistical analysis included the calculation of the ROR, IC, and EBGM, with their respective lower and upper confidence limits. The integration of three statistical methods allowed a comparison of the results to determine their consistency and robustness. A potential signal was considered strong when all measures supported the potential association. Topiramate, a first‐line prophylactic drug for migraine as per several guidelines, was used as the comparator in this study.^17^, ^18^, ^19^ It was identified as a comparator because, among the drugs most recommended for the prevention of chronic migraine, it was the first to be authorized for this therapeutic indication, and thus with the largest postmarketing evidence available. We performed a disproportionality analysis using the ROR, with 95% CI, as statistical parameter to evaluate drug‐adverse drug reaction pairs distribution. ROR is a measure that allows a quantitative approach using 2 × 2 contingency tables, comparing the odds of a particular AE occurring with mAbs to the odds of it occurring with the comparator drug in the database. A ROR > 1 indicates an increased frequency for the drug–ADR pair. The EMA provides guidance on signal detection and management in pharmacovigilance to define signals of disproportionate reporting in the EV system, and these criteria were employed in this study. The criteria applied were as follows: a lower bound of the 95% CI > 1; number of individual cases of three or more for active substances in medicinal products on the additional monitoring list according to the Article 4.1.3 of the Regulation (as per Guideline on good pharmacovigilance practices ‐ GVP ‐ Module X), unless inclusion on the list was solely due to a request for a postauthorization safety study; five individual cases for other active substances; and the event must be listed as an IME.^20^

The IC method is a statistical approach based on Bayesian data mining, measuring disproportionality by comparing observed and expected frequencies of drug–ADR pairs. Positive IC values suggest a higher‐than‐expected frequency of AEs, implying a potential signal, whereas negative IC values suggest a lower‐than‐expected frequency. IC025, the lower limit of the 95% credibility interval, is used to assess signal strength. It provides a threshold that must be exceeded to consider an association signal positive, ensuring that the detected signal is not due to random variation. A positive IC025 indicates a statistically significant association.

The EBGM, another Bayesian data mining approach, also measures disproportionality by comparing observed and expected frequencies of drug–ADR pairs. The strength and reliability of the signal are assessed using the lower and upper bounds of the 95% credibility interval, EB05, and EB95, respectively. A high EB05 value indicates a statistically significant signal, suggesting a higher‐than‐expected frequency of reports for the drug–ADR pair. The disproportionality analysis methods are summarized in Table 2.

**TABLE 2:** Disproportionality analysis methods.

Disproportionality analysis methods	Formula	Criteria
ROR	$\mathrm{ROR}=\frac{a\times d}{b\times c}$ $95\%\mathrm{CI}=e^{\ln\left(\mathrm{ROR}\right)\mp 1.96\times \sqrt{\left(\frac{1}{a}+\frac{1}{b}+\frac{1}{c}+\frac{1}{d}\right)}}$	ROR > 1 Lower limit of 95% CI > 1
IC	$\mathrm{IC}=\log_{2}\;\frac{a\times \left(a+b+c+d\right)}{\left(a+c\right)\times \left(a+b\right)}$ $95\%\mathrm{CI}=E\left(\mathrm{IC}\right)\mp 1.96\sqrt{V\left(\mathrm{IC}\right)}$	IC025 > 0
EBGM	$\text{EBGM}=\frac{a\times \left(a+b+c+d\right)}{\left(a+c\right)\times \left(a+b\right)}$ $95\%\mathrm{CI}=e^{\ln\left(\text{EBGM}\right)\mp 1.96\times \sqrt{\left(\frac{1}{a}+\frac{1}{b}+\frac{1}{c}+\frac{1}{d}\right)}}$	EBGM05 > 2

*Note*: *a*, number of reports of the specific adverse reaction for the drug of interest; *b*, number of reports of other adverse reactions for the same drug; *c*, number of reports of the same adverse reaction for other drugs; *d*, number of reports of other adverse reactions for other drugs.

Abbreviations: CI, confidence interval; E(IC), IC expectations; EBGM, empirical Bayes geometric mean; EBGM05, lower limit of 95% CI of EBGM; IC, information component; IC025, the lower limit of 95% CI of the IC; ROR, reporting odds ratio; V(IC), variance of IC.

The significance level (alpha) was set at 0.05, and all statistical tests were two‐sided.

The Reporting of a Disproportionality Analysis for Drug Safety Signal Detection Using Individual Case Safety Reports in PharmacoVigilance guideline was used.^21^ An institutional review board statement is not applicable.

## RESULTS

### Descriptive

**FIGURE 1:**
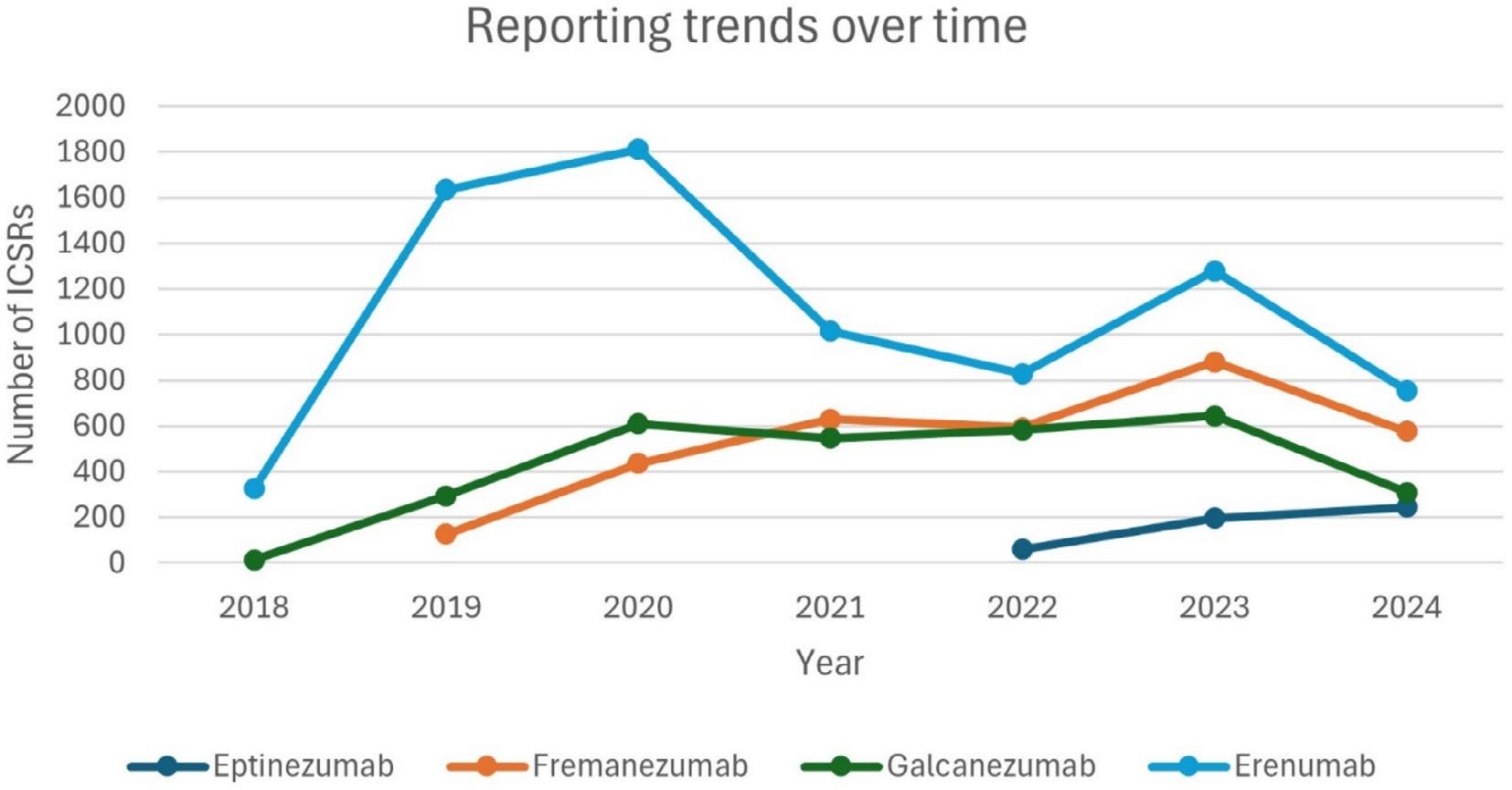
Number of Individual Case Safety Reports (ICSRs) for eptinezumab, erenumab, fremanezumab, and galcanezumab from their date of first approval until June 16, 2024. [Colour figure can be viewed at wileyonlinelibrary.com]

Figure 1 shows the reporting trend over time for eptinezumab, erenumab, fremanezumab, and galcanezumab, whereas Table 3 summarizes the characteristics of each report by patients' sex (female, male, and unknown), age range (0–17 years, 18–64 years, 65–85 years, >85 years, and unknown), and type of reporter (health care professional and non‐health care professional). A total of 14,285 reports were collected for these products: 501 ICSRs for eptinezumab, 7547 for erenumab, 3241 for fremanezumab, and 2996 for galcanezumab. The proportions of reports for females were higher than for males, with similar percentages among all mAbs: 83.8% versus 12.8% for eptinezumab, 86.7% versus 11.2% for erenumab, 88.7% versus 8.5% for fremanezumab, and 83.6 versus 11.1% for galcanezumab. However, in 2.9% reports out of the total, the information on sex was missing. Most of the reports referred to patients aged 18–64 years, a small percentage referred to pediatric populations and those >85 years old, whereas nearly 34% of the reports were without information on the age of the patient. A percentage of 65.2% of ICSRs were submitted by health care professionals.

**TABLE 3:** Demographic characteristics and reporter type for reports of eptinezumab, erenumab, fremanezumab, and galcanezumab distributed by the number of ICSRs (%).

Characteristics	Monoclonal antibodies			
	Eptinezumab, *n* = 501	Erenumab, *n* = 7547	Fremanezumab, *n* = 3241	Galcanezumab, *n* = 2996
Sex				
Female	420 (83.8)	6547 (86.7)	2876 (88.7)	2504 (83.6)
Male	64 (12.8)	843 (11.2)	277 (8.5)	334 (11.1)
Unknown	17 (3.4)	157 (2.1)	88 (2.7)	158 (5.3)
Age in years				
0–17	1 (0.2)	28 (0.4)	8 (0.2)	10 (0.3)
18–64	376 (75.0)	4030 (53.4)	2275 (70.2)	1852 (61.8)
65–85	36 (7.2)	428 (5.7)	163 (5.0)	198 (6.6)
>85	1 (0.2)	13 (0.2)	7 (0.2)	7 (0.2)
Unknown	87 (17.4)	3048 (40.4)	788 (24.3)	929 (31.0)
Reporter type				
Health care professional	336 (67.1)	5343 (70.8)	1861 (57.4)	1767 (59.0)
Non‐health care professional	165 (32.9)	2204 (29.2)	1380 (42.6)	1229 (41.0)

### Disproportionality analysis

For the disproportionality analysis, all 14,285 ICSRs had been examined, corresponding to 46,278 drug–ADR pairs distributed as follows: 1333 PTs for eptinezumab, 26,342 for erenumab, 10,511 for fremanezumab, and 8092 for galcanezumab. We did not consider reported ADRs referring to incorrect product storage, routine laboratory tests, or incorrect administration, as they were not pertinent to our investigation. All statistically significant PTs are listed in the respective tables (see Tables 4, 5, 6, 7), and the three most frequent PTs and the three PTs with the highest ROR for each mAb are listed below with their statistical parameters.

**TABLE 4:** Most frequent and statistically significant ADRs related to eptinezumab.

ADR	*n*	n*	ROR (95% CI)	IC	IC025	EBGM	EB05
Migraine	**78**	**197**	**3.94 (3.01–5.15)**	**1.58**	**1.31**	**2.98**	**2.28**
Fatigue	**51**	**141**	**3.54 (2.56–4.90)**	**1.48**	**1.16**	**2.79**	**2.02**
Headache	44	197	2.16 (1.55–3.02)	0.94	0.61	1.92	1.38
Nausea	32	176	1.75 (1.19–2.56)	0.69	0.31	1.62	1.11
Anaphylactic reaction	**26**	**6**	**42.05 (17.27–102.34)**	**3.10**	**2.21**	**8.55**	**3.51**
Pruritus	**26**	**22**	**11.45 (6.47–20.26)**	**2.51**	**1.94**	**5.70**	**3.22**
Hypersensitivity	20	46	4.19 (2.47–7.10)	1.67	1.14	3.19	1.88
Dyspnea	20	63	3.05 (1.84–5.06)	1.34	0.84	2.53	1.53
Rash	18	51	3.39 (1.98–5.82)	1.46	0.92	2.74	1.60
Cough	16	35	4.39 (2.42–7.96)	1.72	1.13	3.30	1.82
Throat irritation	**15**	**4**	**36.09 (11.96–108.90)**	**3.05**	**1.95**	**8.30**	**2.75**
COVID‐19	**15**	**15**	**9.62 (4.69–19.71)**	**2.39**	**1.68**	**5.26**	**2.57**
Nasopharyngitis	**13**	**12**	**10.40 (4.74–22.85)**	**2.45**	**1.66**	**5.47**	**2.49**
Palpitations	**13**	**18**	**6.93 (3.39–14.18)**	**2.14**	**1.43**	**4.41**	**2.16**
Constipation	13	34	3.67 (1.93–6.96)	1.54	0.90	2.91	1.53
Oropharyngeal pain	**12**	**16**	**7.19 (3.40–15.24)**	**2.17**	**1.42**	**4.51**	**2.13**
Nasal congestion	**10**	**24**	**15.98 (5.80–44.03)**	**2.72**	**1.70**	**6.57**	**2.39**
Vertigo	10	6	3.99 (1.90–8.36)	1.63	0.89	3.09	1.48
Loss of consciousness	10	28	3.42 (1.66–7.05)	1.47	0.74	2.77	1.34
Erythema	**9**	**7**	**12.31 (4.58–33.12)**	**2.56**	**1.58**	**5.92**	**2.20**
Urticaria	8	31	2.47 (1.13–5.37)	1.11	0.33	2.16	0.99
Drug effect less than expected	7	2	33.48 (6.95–161.36)	3.03	1.46	8.18	1.70
Influenzalike illness	7	4	16.74 (4.89–57.26)	2.74	1.51	6.69	1.96
Rhinorrhea	7	4	16.74 (4.89–57.26)	2.74	1.51	6.69	1.96
Blood pressure increased	7	6	11.16 (3.74–33.25)	2.50	1.41	5.66	1.90
Infusion‐related reaction	7	22	3.04 (1.30–7.13)	1.34	0.49	2.54	1.08
Paresthesia, oral	6	8	7.17 (2.48–20.69)	2.17	1.11	4.51	1.56
Sinusitis	6	8	7.17 (2.48–20.69)	2.17	1.11	4.51	1.56
Chest pain	6	21	2.73 (1.10–6.77)	1.22	0.32	2.34	0.94
Feeling hot	5	3	15.92 (3.80–66.69)	2.72	1.28	6.57	1.57
Presyncope	5	6	7.96 (2.43–26.11)	2.26	1.07	4.78	1.46
Neuropathy peripheral	5	7	6.82 (2.16–21.52)	2.13	0.98	4.38	1.39
Syncope	5	10	4.77 (1.63–13.99)	1.81	0.73	3.51	1.20
Asthma	5	14	3.41 (1.23–9.48)	1.47	0.45	2.77	1.00
Bronchospasm	4	1	38.19 (4.26–341.90)	3.07	0.88	8.41	0.94
Dry skin	4	3	12.73 (2.85–56.92)	2.59	1.09	6.01	1.34
Pharyngeal swelling	4	3	12.73 (2.85–56.92)	2.59	1.09	6.01	1.34
Chest discomfort	4	4	9.54 (2.38–38.21)	2.39	1.01	5.26	1.31
Blood pressure decreased	4	5	7.63 (2.05–28.47)	2.22	0.91	4.67	1.25
Flushing	4	7	5.45 (1.59–18.65)	1.94	0.71	3.82	1.12
Arrhythmia	4	8	4.77 (1.43–15.86)	1.81	0.61	3.51	1.05
Tachycardia	4	8	4.77 (1.43–15.86)	1.81	0.61	3.51	1.05
Therapeutic response decreased	4	8	4.77 (1.43–15.86)	1.81	0.61	3.51	1.05
Feeling cold	4	9	4.24 (1.30–13.79)	1.69	0.52	3.24	1.00
Product dose omission issue	4	10	3.82 (1.20–12.18)	1.59	0.43	3.01	0.94
Illness	4	11	3.47 (1.10–10.91)	1.49	0.34	2.80	0.89
Cluster headache	3	1	14.31 (2.39–85.70)	2.66	0.87	6.31	1.05
Throat tightness	3	2	14.31 (2.39–85.70)	2.66	0.87	6.31	1.05
Erectile dysfunction	3	2	28.62 (2.97–275.32)	2.98	0.72	7.89	0.82
Diverticulitis	3	4	7.15 (1.60–31.99)	2.17	0.67	4.51	1.01
Surgery	3	4	7.15 (1.60–31.99)	2.17	0.67	4.51	1.01
Abortion, spontaneous	3	5	5.72 (1.37–23.97)	1.98	0.55	3.94	0.94
Ear pain	3	5	5.72 (1.37–23.97)	1.98	0.55	3.94	0.94
Heart rate decreased	3	5	5.72 (1.37–23.97)	1.98	0.55	3.94	0.94
Herpes zoster	3	6	4.77 (1.19–19.09)	1.81	0.42	3.51	0.88
Intestinal obstruction	3	6	4.77 (1.19–19.09)	1.81	0.42	3.51	0.88
Pregnancy	3	6	4.77 (1.19–19.09)	1.81	0.42	3.51	0.88
Sensory disturbance	3	7	4.09 (1.06–15.82)	1.66	0.30	3.16	0.82

*Note:* Boldface indicates Preferred Terms conforming to the four signal detection algorithms.

Abbreviations: ADR, adverse drug reaction; CI, confidence interval; EB05, lower limit of the 95% CI of the EBGM; EBGM, empirical Bayesian geometric mean; IC, information component; IC025, lower limit of the 95% CI of the IC; *n*, number of suspected ADRs; n*, number of suspected ADRs to topiramate; ROR, reporting odds ratio.

**TABLE 5:** Most frequent and statistically significant ADRs related to erenumab.

ADR	*n*	n*	ROR (95% CI)	IC	IC025	EBGM	EB05
Migraine	1126	578	2.04 (1.85–2.26)	0.42	0.32	1.34	1.21
Constipation	1027	61	17.94 (13.85–23.24)	0.94	0.68	1.91	1.48
Fatigue	532	312	1.77 (1.53–2.03)	0.35	0.21	1.28	1.11
Nausea	494	336	1.52 (1.32–1.75)	0.27	0.13	1.21	1.05
Alopecia	469	162	3.01 (2.51–3.60)	0.59	0.41	1.51	1.26
Arthralgia	327	124	2.73 (2.22–3.36)	0.55	0.35	1.47	1.19
Pruritus	294	69	4.41 (3.39–5.74)	0.71	0.45	1.64	1.26
Injection site pain	213	21	10.48 (6.69–16.42)	0.88	0.43	1.84	1.18
Muscle spasms	212	51	4.29 (3.16–5.83)	0.71	0.40	1.63	1.20
Myalgia	211	47	4.64 (3.38–6.36)	0.73	0.41	1.66	1.21
Weight increased	199	113	1.81 (1.44–2.29)	0.37	0.14	1.29	1.03
Wrong technique in product usage process	197	36	5.65 (3.96–8.06)	0.78	0.42	1.71	1.20
Dyspnea	179	126	1.46 (1.16–1.84)	0.25	0.02	1.19	0.95
Rash	173	108	1.65 (1.30–2.10)	0.32	0.08	1.25	0.98
Hypertension	167	53	3.25 (2.38–4.43)	0.62	0.31	1.54	1.13
Abdominal pain	157	96	1.68 (1.30–2.17)	0.33	0.08	1.26	0.97
Palpitations	133	44	3.11 (2.21–4.38)	0.61	0.27	1.52	1.08
Abdominal distension	132	58	2.34 (1.72–3.19)	0.49	0.18	1.41	1.03
Urticaria	129	54	2.46 (1.79–3.38)	0.51	0.20	1.43	1.04
Malaise	126	82	1.58 (1.20–2.09)	0.30	0.02	1.23	0.93
Dyspepsia	122	64	1.96 (1.45–2.65)	0.41	0.11	1.33	0.98
Pain in extremity	119	55	2.23 (1.62–3.07)	0.47	0.15	1.39	1.01
Tinnitus	106	62	1.76 (1.28–2.41)	0.35	0.04	1.28	0.93
Erythema	98	24	4.20 (2.69–6.57)	0.70	0.26	1.63	1.04
Sleep disorder	97	59	1.69 (1.22–2.34)	0.33	0.01	1.26	0.91
Therapeutic product effect decreased	96	42	2.35 (1.63–3.38)	0.50	0.13	1.41	0.98
Blood pressure increased	91	16	5.85 (3.44–9.96)	0.79	0.25	1.72	1.01
Chest pain	90	40	2.31 (1.59–3.36)	0.49	0.12	1.40	0.97
Inappropriate schedule of product administration	85	26	3.36 (2.17–5.22)	0.63	0.19	1.55	1.00
Influenzalike illness	81	10	8.33 (4.32–16.08)	0.85	0.19	1.80	0.93
Cerebrovascular accident	76	31	2.52 (1.67–3.83)	0.53	0.11	1.44	0.95
COVID‐19	74	17	4.48 (2.64–7.59)	0.72	0.19	1.65	0.97
Incorrect dose administered	73	28	2.68 (1.73–4.14)	0.55	0.11	1.46	0.95
Nasopharyngitis	73	24	3.13 (1.97–4.96)	0.61	0.15	1.52	0.96
Product storage error	65	6	11.14 (4.83–25.72)	0.89	0.05	1.85	0.80
Injection site reaction	63	20	3.24 (1.96–5.36)	0.62	0.12	1.54	0.93
Contusion	59	24	2.53 (1.57–4.06)	0.53	0.05	1.44	0.90
Chest discomfort	58	12	4.97 (2.67–9.25)	0.75	0.13	1.68	0.90
Incorrect route of product administration	54	20	2.77 (1.66–4.64)	0.56	0.05	1.48	0.88
Myocardial infarction	46	13	3.64 (1.96–6.73)	0.66	0.04	1.58	0.85
Flushing	42	11	3.92 (2.02–7.62)	0.68	0.02	1.61	0.83
Poor quality sleep	42	10	4.32 (2.16–8.60)	0.71	0.02	1.64	0.82

Abbreviations: ADR, adverse drug reaction; CI, confidence interval; EB05, lower limit of the 95% CI of the EBGM; EBGM, empirical Bayesian geometric mean; IC, information component; IC025, lower limit of the 95% CI of the IC; *n*, number of suspected ADRs; n*, number of suspected ADRs to topiramate; ROR, reporting odds ratio.

**TABLE 6:** Most frequent and statistically significant ADRs related to fremanezumab.

ADR	*n*	n*	ROR (95% CI)	IC	IC025	EBGM	EB05
Injection site swelling	186	2	222.62 (55.25–896.98)	1.73	0.34	3.32	0.82
Pruritus	185	57	7.75 (5.75–10.44)	1.36	1.06	2.56	1.90
Nausea	179	308	1.37 (1.14–1.65)	0.30	0.12	1.23	1.02
Weight increased	157	104	3.59 (2.80–4.60)	1.01	0.76	2.02	1.57
Myalgia	113	44	6.09 (4.30–8.64)	1.27	0.92	2.41	1.70
Urticaria	80	50	3.78 (2.66–5.39)	1.04	0.69	2.06	1.45
Injection site reaction	73	20	8.64 (5.26–14.17)	1.40	0.90	2.63	1.60
Abdominal pain	61	85	1.69 (1.22–2.35)	0.49	0.16	1.40	1.01
Hypertension	59	49	2.84 (1.94–4.15)	0.87	0.49	1.83	1.25
Swelling	46	12	9.05 (4.79–17.09)	1.41	0.77	2.66	1.41
Abdominal distension	46	58	1.87 (1.27–2.75)	0.57	0.18	1.48	1.01
Pyrexia	43	60	1.69 (1.14–2.50)	0.48	0.09	1.40	0.95
Tinnitus	38	56	1.60 (1.06–2.41)	0.44	0.03	1.35	0.90
Heart rate increased	37	28	3.11 (1.91–5.09)	0.93	0.44	1.91	1.17
Peripheral swelling	36	31	2.74 (1.69–4.43)	0.85	0.37	1.80	1.11
Vertigo	36	50	1.70 (1.10–2.60)	0.49	0.06	1.40	0.91
Injection site mass	34	2	40.10 (9.63–166.96)	1.66	0.24	3.17	0.76
Cerebrovascular accident	29	31	2.20 (1.33–3.66)	0.70	0.19	1.62	0.98
Musculoskeletal stiffness	25	28	2.10 (1.23–3.61)	0.66	0.12	1.58	0.92
Raynaud's phenomenon	24	3	18.85 (5.68–62.63)	1.57	0.37	2.98	0.90
Needle issue	20	2	23.56 (5.51–100.81)	1.61	0.15	3.05	0.71
Edema	19	2	22.38 (5.21–96.10)	1.60	0.14	3.03	0.71
Chills	19	20	2.24 (1.19–4.19)	0.71	0.08	1.63	0.87
Dry eye	18	19	2.23 (1.17–4.25)	0.71	0.06	1.63	0.86
Menstruation irregular	16	6	6.28 (2.46–16.05)	1.29	0.35	2.44	0.95
Dry skin	16	9	4.19 (1.85–9.47)	1.10	0.28	2.15	0.95
Arrhythmia	16	17	2.22 (1.12–4.39)	0.70	0.02	1.63	0.82
Acne	13	10	3.06 (1.34–6.98)	0.92	0.10	1.89	0.83
Flushing	13	11	2.78 (1.25–6.21)	0.86	0.06	1.82	0.81
Night sweats	11	7	3.70 (1.43–9.54)	1.03	0.09	2.05	0.79
Initial insomnia	11	9	2.88 (1.19–6.94)	0.88	0.00	1.84	0.76
Increased appetite	9	4	5.29 (1.63–17.20)	1.21	0.04	2.32	0.71

Abbreviations: ADR, adverse drug reaction; CI, confidence interval; EB05, lower limit of the 95% CI of the EBGM; EBGM, empirical Bayesian geometric mean; IC, information component; IC025, lower limit of the 95% CI of the IC; *n*, number of suspected ADRs; n*, number of suspected ADRs to topiramate; ROR, reporting odds ratio.

#### Eptinezumab

Eptinezumab versus topiramate results were as follows: migraine, *n* = 78 versus 197 events (ROR = 3.94 [95% CI = 3.01–5.15], IC = 1.58 [IC025 = 1.31], EBGM = 2.98 [EB05 = 2.28]); fatigue, *n* = 51 versus 141 events (ROR = 3.54 [95% CI = 2.56–4.90], IC = 1.48 [IC025 = 1.16], EBGM = 2.79 [EB05 = 2.02]); headache, *n* = 44 versus 197 events (ROR = 2.16 [95% CI = 1.55–3.02], IC = 0.94 [IC025 = 0.61], EBGM = 1.92 [EB05 = 1.38]). The three AEs with all the highest statistical parameters were anaphylactic reaction (ROR = 42.05 [95% CI = 17.27–102.34], *n* = 26 vs. 6 events, IC = 3.10 [IC025 = 2.21], EBGM = 8.55 [EB05 3.51]), bronchospasm (ROR = 38.19 [95% CI = 4.26–341.90], *n* = 4 vs. 1 events, IC = 3.07 [IC025 = 0.88], EBGM = 8.41 [EB05 = 0.94]), and throat irritation (ROR = 36.09 [95% CI = 11.96–108.90], *n* = 15 vs. 4 events, IC = 3.05 [IC025 = 1.95], EBGM = 8.30 [EB05 = 2.75]).

#### Erenumab

Erenumab versus topiramate results were as follows: migraine, *n* = 1126 versus 578 events (ROR = 2.04 [95% CI = 1.85–2.26], IC = 0.42 [IC025 = 0.32], EBGM = 1.34 [EB05 = 1.21]); constipation, *n* = 1027 versus 61 events (ROR = 17.94 [95% CI = 13.85–23.24], IC = 0.94 [IC025 = 0.68], EBGM = 1.91 [EB05 = 1.48]); fatigue, *n* = 532 versus 312 events (ROR = 1.77 [95% CI = 1.53–2.03], IC = 0.35 [IC025 = 0.21], EBGM = 1.28 [EB05 = 1.11]. The three AEs with all three highest statistical measures were constipation (ROR = 17.94 [95% CI = 13.85–23.24], *n* = 1027 vs. 61 events, IC = 0.94 [IC025 = 0.68], EBGM = 1.91 [EB05 = 1.48]), injection site pain (ROR = 10.48 [95% CI = 6.69–16.42], *n* = 213 vs. 21 events, IC = 0.88 [IC025 = 0.43], EBGM = 1.84 [EB05 = 1.18]), and influenza like illness (ROR = 8.33 [95% CI = 4.32–16.08], *n* = 81 vs. 10 events, IC = 0.85 [IC025 = 0.19], EBGM = 1.80 [EB05 = 0.93]).

#### Fremanezumab

Fremanezumab versus topiramate results were as follows: injection site swelling, *n* = 186 versus 2 events (ROR = 222.62 [95% CI = 55.25–896.98], IC = 1.73 [IC025 = 0.34], EBGM = 3.32 [EB05 = 0.82]); pruritus, *n* = 185 versus 57 events (ROR = 7.75 [95% CI = 5.75–10.44], IC = 1.36 [IC025 = 1.06], EBGM = 2.56 [EB05 = 1.90]); nausea, *n* = 179 versus 308 events (ROR = 1.37 [95% CI = 1.14–1.65], IC = 0.30 [IC025 = 0.12], EBGM = 1.23 [EB05 = 1.02]). The three AEs with all the highest statistical parameters were injection site swelling (ROR = 222.62 [95% CI = 55.25–896.98], *n* = 186 vs. 2 events, IC = 1.73 [IC025 = 0.34], EBGM = 3.32 [EB05 = 0.82]), injection site mass (ROR = 40.10 [95% CI = 9.63–166.96], *n* = 34 vs. 2 events, IC = 1.66 [IC025 = 0.24], EBGM = 3.17 [EB05 = 0.76]), and edema (ROR = 22.38 [95% CI = 5.21–96.10], *n* = 19 vs. 2 events, IC = 1.60 [IC025 = 0.14], EBGM = 3.03 [EB05 = 0.71]).

#### Galcanezumab

Galcanezumab versus topiramate results were as follows: migraine, *n* = 257 versus 559 events (ROR = 1.51 [95% CI = 1.30–1.75], IC = 0.42 [IC025 = 0.27], EBGM = 1.34 [EB05 = 1.15]); injection site pain, *n* = 214 versus 21 events (ROR = 33.90 [95% CI = 21.64–53.11], IC = 1.95 [IC025 = 1.50], EBGM = 3.86 [EB05 = 2.47]), constipation, *n* = 195 versus 60 events (ROR = 10.77 [95% CI = 8.06–14.40], IC = 1.70 [IC025 = 1.41], EBGM = 3.24 [EB05 = 2.43]). The three AEs with all the highest statistical measures were injection site erythema (ROR = 576.41 [95% CI = 80.72–4115.87], *n* = 174 vs. 1 events, IC = 2.08 [IC025 = 0.11], EBGM = 4.22 [EB05 = 0.59]), injection site swelling (ROR = 149.16 [95% CI = 36.73–605.66], *n* = 91 vs. 2 events, IC = 2.05 [IC025 = 0.65], EBGM =4.15 [EB05 = 1.02]), and injection site warmth (ROR = 97.61 [95% CI = 13.31–715.87], *n* = 30 vs. 1 events, IC = 2.04 [IC025 = 0.04], EBGM = 4.10 [EB05 = 0.56]).

Considering the strengths of the associations, a total of 15 significant disproportionality PTs conforming to the four signal detection algorithms were obtained: 11 for eptinezumab, four for galcanezumab, and none for fremanezumab and erenumab. According to the IME list, among these there is only one serious PT, which is the already known anaphylactic reaction for eptinezumab. Of these, only eptinezumab showed five potential new safety signals: migraine, *n* = 78 versus 197 events (ROR = 3.94 [95% CI = 3.01–5.15], IC = 1.58 [IC025 = 1.31], EBGM = 2.98 [EB05 = 2.28]); COVID‐19, *n* = 15 versus 15 events (ROR = 9.62 [95% CI = 4.69–19.71], IC = 2.39 [IC025 = 1.68], EBGM = 5.26 [EB05 = 2.57]); palpitations, *n* = 13 versus 18 events (ROR = 6.93 [95% CI = 3.39–14.18], IC = 2.14 [IC025 = 1.43], EBGM = 4.41 [EB05 = 2.16]); oropharyngeal pain, *n* = 12 versus 16 events (ROR = 7.19 [95% CI = 3.40–15.24], IC = 2.17 [IC025 = 1.42], EBGM = 4.51 [EB05 = 2.13]); erythema, *n* = 9 versus 7 events (ROR = 12.31 [95% CI = 4.58–33.12], IC = 2.56 [IC025 = 1.58], EBGM = 5.92 [EB05 = 2.20]). The detailed data for all these 15 PTs are shown in Tables 4 and 7, highlighted in bold.

**TABLE 7:** Most frequent and statistically significant ADRs related to galcanezumab.

ADR	*n*	n*	ROR (95% CI)	IC	IC025	EBGM	EB05
Migraine	257	559	1.51 (1.30–1.75)	0.42	0.27	1.34	1.15
Injection site pain	**214**	**21**	**33.90 (21.64–53.11)**	**1.95**	**1.50**	**3.86**	**2.47**
Constipation	**195**	**60**	**10.77 (8.06–14.40)**	**1.70**	**1.41**	**3.24**	**2.43**
Injection site erythema	174	1	576.41 (80.72–4115.87)	2.08	0.11	4.22	0.59
Dizziness	160	299	1.75 (1.44–2.12)	0.56	0.37	1.48	1.22
Pruritus	**155**	**66**	**7.74 (5.80–10.34)**	**1.57**	**1.28**	**2.97**	**2.23**
Fatigue	145	300	1.58 (1.29–1.93)	0.47	0.27	1.38	1.13
Alopecia	130	156	2.73 (2.16–3.45)	0.95	0.71	1.93	1.53
Injection site pruritus	111	5	72.95 (29.77–178.79)	2.02	1.12	4.06	1.66
Weight increased	110	112	3.21 (2.47–4.19)	1.07	0.81	2.10	1.61
Arthralgia	109	124	2.87 (2.22–3.72)	0.99	0.73	1.98	1.53
Injection site swelling	91	2	149.16 (36.73–605.66)	2.05	0.65	4.15	1.02
Dyspnea	85	120	2.31 (1.75–3.05)	0.81	0.54	1.76	1.33
Rash	84	102	2.69 (2.01–3.59)	0.94	0.65	1.92	1.43
Hypersensitivity	81	70	3.78 (2.74–5.21)	1.19	0.87	2.28	1.65
Myalgia	74	47	5.14 (3.56–7.42)	1.38	1.01	2.59	1.80
Injection site reaction	**73**	**20**	**11.93 (7.27–19.58)**	**1.74**	**1.24**	**3.33**	**2.03**
Anxiety	69	148	1.52 (1.14–2.02)	0.43	0.14	1.35	1.01
Urticaria	62	53	3.81 (7.64–5.21)	1.19	0.83	2.29	1.58
Insomnia	62	134	1.50 (1.11–2.03)	0.42	0.12	1.34	0.99
Product dose omission issue	56	43	4.24 (2.85–6.32)	1.26	0.86	2.40	1.61
Vertigo	56	56	3.26 (2.25–4.72)	1.08	0.71	2.12	1.46
Anaphylactic reaction	55	15	11.96 (6.75–21.18)	1.74	1.17	3.33	1.88
Underdose	50	9	18.11 (8.90–36.85)	1.85	1.14	3.59	1.77
Palpitations	47	44	3.48 (2.30–5.25)	1.13	0.72	2.19	1.45
Cerebrovascular accident	45	31	4.73 (2.99–7.47)	1.33	0.87	2.51	1.59
Hypertension	44	52	2.75 (1.84–4.12)	0.96	0.56	1.94	1.30
Chest pain	39	39	3.25 (2.09–5.07)	1.08	0.64	2.12	1.36
Loss of consciousness	39	60	2.11 (1.41–3.16)	0.74	0.34	1.67	1.12
Feeling abnormal	37	66	1.82 (1.22–2.73)	0.61	0.20	1.52	1.02
Blood pressure increased	35	16	7.12 (3.94–12.87)	1.54	0.95	2.91	1.61
COVID‐19	35	17	6.70 (3.75–11.96)	1.51	0.93	2.85	1.60
Erythema	35	23	4.95 (2.92–8.38)	1.36	0.83	2.56	1.51
Injection site warmth	30	1	97.61 (13.3–715.87)	2.04	0.04	4.10	0.56
Tinnitus	30	58	1.68 (1.08–2.61)	0.53	0.09	1.45	0.93
Incorrect dose administered	29	26	3.63 (2.13–6.16)	1.16	0.63	2.24	1.32
Myocardial infarction	27	13	6.75 (3.48–13.09)	1.52	0.86	2.86	1.48
Syncope	27	31	2.83 (1.69–4.74)	0.98	0.46	1.97	1.18
Angioedema	27	32	2.74 (1.64–4.58)	0.96	0.44	1.94	1.16
Nightmare	27	39	2.25 (1.38–3.67)	0.80	0.30	1.74	1.06
Hyperhidrosis	27	43	2.04 (1.26–3.30)	0.71	0.23	1.64	1.01
Chest discomfort	26	11	7.68 (3.79–15.56)	1.58	0.87	2.98	1.47
Raynaud's phenomenon	25	3	27.09 (8.18–89.76)	1.92	0.72	3.79	1.14
Illness	25	24	3.38 (1.93–5.93)	1.11	0.55	2.16	1.24
Injection site hemorrhage	24	2	39.01 (9.22–165.11)	1.97	0.53	3.92	0.93
Heart rate increased	24	28	2.78 (1.61–4.80)	0.97	0.42	1.96	1.13
Injection site mass	23	2	37.38 (8.81–158.59)	1.96	0.52	3.90	0.92
Inappropriate schedule of product administration	22	25	2.86 (1.61–5.07)	0.99	0.42	1.99	1.12
Abortion, spontaneous	22	31	2.30 (1.33–3.98)	0.82	0.27	1.76	1.02
Swelling	21	15	4.55 (2.34–8.83)	1.31	0.64	2.47	1.27
Injection site induration	20	1	64.99 (8.72–484.34)	2.01	0.01	4.04	0.54
Tachycardia	20	29	2.24 (1.27–3.96)	0.79	0.22	1.73	0.98
Musculoskeletal stiffness	20	30	2.16 (1.23–3.81)	0.76	0.20	1.70	0.96
Transient ischemic attack	19	5	12.34 (4.61–33.07)	1.75	0.76	3.36	1.25
Hot flush	18	16	3.65 (1.86–7.17)	1.17	0.49	2.25	1.14
Presyncope	17	10	5.52 (2.53–12.06)	1.42	0.64	2.67	1.22
Pharyngeal swelling	16	5	10.39 (3.81–28.38)	1.69	0.69	3.23	1.18
Blindness	16	17	3.05 (1.54–6.05)	1.04	0.36	2.06	1.04
Anaphylactic shock	15	2	24.36 (5.57–106.53)	1.90	0.43	3.74	0.86
Breast cancer	15	2	24.36 (5.57–106.53)	1.90	0.43	3.74	0.86
Chills	15	20	2.43 (1.25–4.76)	0.86	0.19	1.82	0.93
Edema, peripheral	14	10	4.54 (2.02–10.23)	1.31	0.50	2.47	1.10
Brain fog	14	19	2.39 (1.20–4.77)	0.85	0.16	1.80	0.90
Throat tightness	13	4	10.55 (3.44–32.37)	1.70	0.58	3.24	1.06
Lip swelling	13	6	7.03 (2.67–18.51)	1.54	0.57	2.90	1.10
Edema	13	6	7.03 (2.67–18.51)	1.54	0.57	2.90	1.10
Feeling hot	13	8	5.27 (2.19–12.73)	1.39	0.51	2.63	1.09
Blood pressure decreased	13	11	3.84 (1.72–8.56)	1.20	0.40	2.30	1.03
Road traffic accident	13	12	3.52 (1.60–7.71)	1.14	0.36	2.21	1.01
Restless legs syndrome	13	18	2.34 (1.15–4.78)	0.83	0.12	1.78	0.87
Brain neoplasm	12	2	19.48 (4.36–87.04)	1.86	0.37	3.64	0.81
Abnormal dreams	12	8	4.87 (1.99–11.91)	1.35	0.45	2.54	1.04
Injection site inflammation	11	2	17.85 (3.96–80.56)	1.84	0.34	3.59	0.80
Pancreatitis	11	6	5.95 (2.20–16.09)	1.46	0.46	2.74	1.01
Atrial fibrillation	11	8	4.46 (1.79–11.10)	1.30	0.39	2.46	0.99
Rash, pruritic	11	8	4.46 (1.79–11.10)	1.30	0.39	2.46	0.99
Influenzalike illness	11	9	3.97 (1.64–9.57)	1.22	0.34	2.33	0.97
Poor‐quality sleep	11	10	3.57 (1.52–8.41)	1.15	0.29	2.22	0.94
Flatulence	10	5	6.49 (2.22–18.99)	1.50	0.43	2.83	0.97
Head injury	10	8	4.06 (1.60–10.28)	1.24	0.31	2.36	0.93
Pulmonary embolism	10	10	3.24 (1.35–7.80)	1.08	0.21	2.12	0.88
Skin reaction	9	3	9.73 (2.63–35.97)	1.67	0.36	3.18	0.86
Aura	9	6	4.87 (1.73–13.68)	1.35	0.31	2.54	0.91
Heavy menstrual bleeding	9	6	4.87 (1.73–13.68)	1.35	0.31	2.54	0.91
Product storage error	9	6	4.87 (1.73–13.68)	1.35	0.31	2.54	0.91
Cluster headache	9	7	4.17 (1.55–11.20)	1.25	0.27	2.39	0.89
Herpes zoster	9	7	4.17 (1.55–11.20)	1.25	0.27	2.39	0.89
Rash erythematous	8	2	12.98 (2.76–61.13)	1.76	0.21	3.39	0.72
Thrombosis	8	6	4.33 (1.50–12.47)	1.28	0.22	2.42	0.84
Erectile dysfunction	8	8	3.24 (1.22–8.65)	1.08	0.10	2.12	0.80
Dry skin	8	9	2.88 (1.11–7.48)	1.00	0.04	2.00	0.77
Tension headache	8	9	2.88 (1.11–7.48)	1.00	0.04	2.00	0.77
Fluid retention	7	3	7.57 (1.96–29.28)	1.57	0.22	2.97	0.77
Throat irritation	7	5	4.54 (1.44–14.31)	1.31	0.16	2.47	0.79
Menstruation irregular	7	6	3.78 (1.27–11.26)	1.19	0.10	2.28	0.77
Menstrual disorder	7	7	3.24 (1.14–9.25)	1.08	0.04	2.12	0.74
Menstruation delayed	6	3	6.49 (1.62–25.95)	1.50	0.11	2.83	0.71
Increased appetite	6	4	4.87 (1.37–17.25)	1.35	0.08	2.54	0.72
Musculoskeletal discomfort	6	4	4.87 (1.37–17.25)	1.35	0.08	2.54	0.72
Tendonitis	6	2	9.73 (1.96–48.22)	1.67	0.07	3.18	0.64

*Note:* Boldface indicates Preferred Terms conforming to the four signal detection algorithms.

Abbreviations: ADR, adverse drug reaction; CI, confidence interval; EB05, lower limit of the 95% CI of the EBGM; EBGM, empirical Bayesian geometric mean; IC, information component; IC025, lower limit of the 95% CI of the IC; *n*, number of suspected ADRs; n*, number of suspected ADRs to topiramate; ROR, reporting odds ratio.

## DISCUSSION

Although the efficacy and effectiveness of eptinezumab, fremanezumab, galcanezumab, and erenumab in reducing the frequency and severity of migraine attacks has been established, it is essential to understand and monitor the postmarketing safety profiles of these drugs, especially given the chronic nature of migraine treatment. Pharmacovigilance studies based on spontaneous reporting systems, like the present one, are not without limitations. Several limitations are related to the signal‐to‐noise ratio, meaning the ability to distinguish true safety signals from background noise. First, the quality of the data and consequently the reliability of the resulting information are not always excellent, as reports may be incomplete and inaccurate. Confounding factors, such as underlying diseases or concomitant drug use, can influence signal detection. Second, in the absence of the number of patients exposed to a drug, it is not possible to calculate an accurate incidence rate and assess the risk. Moreover, these studies do not include a control group, making it difficult to establish causality between a drug and an ADR. Finally, the system is affected by the underreporting, due to various subjective reasons, above all scarce awareness or lack of time. It is the main intrinsic limitation of spontaneous reporting systems and one of the major drawbacks of pharmacovigilance methods, as it can lead to an underestimation of the frequency of certain ADRs and consequently potential drug‐related risks. In contrast, certain ADRs receive more attention due to, for instance, the media or public influence, leading to overreporting of specific drug‐event pairs.

Furthermore, it is important to take into account the significantly lower number of reports for eptinezumab that may limit the statistical power of the disproportionality analysis, leading to an underestimation of certain ADRs. The smaller number of ICSRs for eptinezumab is likely due to its more recent approval and its intravenous route of administration, which may result in a lower number of treated patients and, consequently, fewer reported ADRs.

The underreporting further affects data validity, especially in ROR estimates.^22^, ^23^, ^24^, ^25^ The use of ROR highlights potential safety signals, without establishing causality. Therefore, integrating it with other methods, such as further disproportionality analyses and observational studies, is essential for a fuller understanding of the safety profile of the medicines. To mitigate these limitations, three other distinct signal detection methods were used in this study, enhancing the reliability of our disproportionality analysis.

The proportion of reports for females was higher than for males among all mAbs, which is in line with several epidemiological studies that have identified differences between sexes in migraine prevalence, with women being affected three times more frequently than men.^26^, ^27^ Moreover, most of the reports referred to patients aged 18–64 years, which is consistent with the disease occurrence.

Our study revealed that overall, almost all of the most reported and statistically significant ADRs for eptinezumab, fremanezumab, galcanezumab, and erenumab were nonserious and consistent with common reactions such as fatigue, pruritus, and AEs related to the injection site. Therefore, the findings confirm the known safety profiles for these medications, aligning with previous clinical trial data and their respective SPCs.

There is one serious PT, which is the anaphylactic reaction for eptinezumab, an event that is already included in the corresponding SPC. Comparing this event to the other mAbs in this study, we observed that no statistically significant suspected anaphylaxis‐related reactions emerged for fremanezumab and erenumab. However, 55 anaphylactic reaction events (ROR = 11.96 [95% CI = 6.75–21.18], IC = 1.74 [IC025 = 1.17], EBGM = 3.33 [EB05 = 1.88]) and 15 anaphylactic shock events (ROR = 24.36 [95% CI = 5.57–106.53], IC = 1.90 [IC025 = 0.43], EBGM = 3.74 [EB05 = 0.86]) were observed for galcanezumab. Although we evaluate all three statistical methods and their wide CIs, the strength of the signals is still weaker. A plausible explanation of this difference could be the different route of administration; whereas fremanezumab, galcanezumab, and erenumab are administered subcutaneously, eptinezumab is administered intravenously, which may increase the risk of immediate hypersensitivity reactions, including anaphylaxis. In addition, as intravenous administrations require controlled environments, operators pay more attention to the patient and are more inclined to report any events that occur during monitoring. As this is a biological drug, variations in amino acid sequences, posttranslational modifications, or excipients could influence the immunogenicity of eptinezumab compared to other mAbs, or even between different batches of the same drug; however, further studies are needed to confirm this hypothesis.

Among the new potential signals detected for eptinezumab, there are palpitations. According to a pooled analysis of five clinical trials conducted by Smith et al., palpitations were the most frequently reported cardiac disorders, which occurred in similar proportions of the overall eptinezumab and placebo arms (8/2076 [0.4%] and 3/791 [0.4%], respectively).^28^ Moreover, the PT palpitations emerged among the reports for erenumab and galcanezumab, showing statistical significance. Other cardiovascular events emerged from our study that have previously required attention, including increased blood pressure, hypertension, and Raynaud's phenomenon (see Tables 4, 5, 6, 7). Our results are consistent with a previous pharmacovigilance analysis conducted on the FDA Adverse Event Reporting System (FAERS) database.^29^ In this regard, a recent systematic review concluded that despite the limited amount of evidence, it is important to monitor blood pressure in patients with migraine.^30^ Furthermore, additional literature supports monitoring of patients for the possibility of new onset or worsening of existing hypertension or Raynaud's phenomenon.^31^, ^32^, ^33^, ^34^, ^35^, ^36^

This evidence could suggest a class effect of anti‐CGRP drugs that warrants further investigation through additional studies. Considering the mechanisms of action outlined in the Risk Management Plan for each anti‐CGRP mAb, the use of these therapies may be associated with the occurrence of certain cardiovascular ADRs. However, as patients with preexisting cardiovascular conditions were excluded from the clinical development program, limited data are available for in use in this population.^37^

The signal of disproportionate reporting for migraine leads to different interpretations. The first is the possibility that it is a reporting error, with migraine representing the target disease for the medicines under study, rather than the actual ADR. Otherwise, it is also possible that reference to the target disease in the context of ADRs may indicate disease progression due to ineffective therapy. Given the high frequency of this PT and its statistically significant measures, this latter interpretation seems more plausible.

It is important to consider the limitations associated with spontaneous reporting, such as potential reporting bias and the quality of information in the reports, which are often not supported by clinical details. Therefore, this signal should be interpreted with caution, and future pharmacoepidemiological studies (such as prospective cohort studies) are needed to further explore these findings, differentiating between therapeutic failure and a possible AE.

Reports from EV have indicated a potential association between the use of eptinezumab and COVID‐19. CGRP plays a role in facilitating an effective immune response against viral infections, although studies on this relationship remain limited.^38^ Moreover, the PROMISE‐1 and PROMISE‐2 trials have not demonstrated a direct increase in viral infections as an adverse effect in patients treated with eptinezumab, suggesting that the drug remains safe with respect to immune system effects.^28^, ^39^

The drug–reaction pair oropharyngeal pain–eptinezumab showed statistical significance and deserves attention. Oropharyngeal pain may be related to the mechanism of action of eptinezumab or to its intravenous administration. Because CGRP is also present in the sensory nerves of the upper airways, including the oropharyngeal region, blocking CGRP might disrupt normal sensory or defensive functions in this area, leading to an abnormal perception of pain or discomfort.^40^ Notably, the same AE emerged from the pharmacovigilance study conducted by Sun et al. on the FAERS database, further reinforcing the signal.^29^ The identification of this AE across both EV and FAERS databases highlights the need to explore whether this is a drug‐specific effect or part of a class effect seen with anti‐CGRP therapies. Given the potential impact on patient safety and adherence to treatment, this finding warrants further investigation.

Lastly, the AE erythema, statistically associated with eptinezumab, has emerged from our analysis. This event, like the previous ones, may be related to the mechanism of action of the mAb, as the CGRP is involved in vasodilation and inflammatory responses and its modulation can potentially lead to vascular changes that contribute to erythema. Additionally, as a biologic therapy, eptinezumab may elicit immune‐mediated reactions, resulting in localized inflammation. Erythema often occurs at the injection site, due to tissue irritation, immune activation, or a direct effect of the drug on blood vessels, leading to increased blood flow and redness.^41^

## CONCLUSIONS

Overall, almost all of the most reported and statistically significant ADRs for eptinezumab, fremanezumab, galcanezumab, and erenumab were nonserious and consistent with the existing literature and respective SPCs. Emerging potential signals such as palpitations, which were observed across multiple CGRP inhibitors, suggest a potential class effect, warranting further investigation. Similarly, oropharyngeal pain associated with eptinezumab might be linked to the role of CGRP in airway sensory function, and erythema could relate to its involvement in vasodilation and inflammation. These findings highlight the importance of assessing long‐term safety, especially as some ADRs, like cardiovascular effects, were underexplored in clinical trials due to the exclusion of patients with preexisting conditions. Despite its limitations, spontaneous reporting systems remain vital for detecting early potential safety signals. The use of additional signal detection methods alongside the ROR enhances the reliability of these observations, but more pharmacoepidemiological studies are needed to clarify the long‐term risks and ensure patient safety in the use of anti‐CGRP therapies. Given the chronic nature of migraine treatment, continuous pharmacovigilance monitoring is essential to ensure the ongoing safety of these products.

## AUTHOR CONTRIBUTIONS

**Victoria Nikitina:** Conceptualization; data curation; formal analysis; investigation; writing – original draft. **Greta Santi Laurini:** Conceptualization; methodology; validation; writing – original draft. **Nicola Montanaro:** Conceptualization; methodology; validation; writing – review and editing. **Domenico Motola:** Conceptualization; investigation; methodology; supervision; writing – review and editing.

## CONFLICT OF INTEREST STATEMENT

**Victoria Nikitina, Greta Santi Laurini, Nicola Montanaro**, and **Domenico Motola** declare no conflicts of interest.

## Data Availability

The data that support the findings of this study are available from the corresponding author upon reasonable request.
